# Improvement of nutritional status after parathyroidectomy in patients receiving maintenance hemodialysis

**DOI:** 10.3389/fmed.2023.1132566

**Published:** 2023-07-06

**Authors:** Sinee Disthabanchong, Sarunya Saeseow, Sirote Khunapornphairote, Ronnarat Suvikapakornkul, Yodying Wasutit, Jumroon Tungkeeratichai

**Affiliations:** ^1^Division of Nephrology, Department of Medicine, Faculty of Medicine, Ramathibodi Hospital, Mahidol University, Bangkok, Thailand; ^2^Department of Surgery, Faculty of Medicine, Ramathibodi Hospital, Mahidol University, Bangkok, Thailand; ^3^Department of Otolaryngology, Faculty of Medicine, Ramathibodi Hospital, Mahidol University, Bangkok, Thailand

**Keywords:** hyperparathyoidism, protein energy wasting, dialysis, parathyroidectomy, sarcopenia, hypoalbuminemia, malnutrition, hypertension

## Abstract

**Aims/Introduction:**

Parathyroidectomy is associated with improved survival in patients with end-stage kidney disease. Protein-energy wasting (PEW) is common in patients with kidney failure and predicts poor outcomes. Recent clinical trials have linked hyperparathyroidism to PEW. The present retrospective cohort study examined whether parathyroidectomy was associated with improvement in nutritional status in maintenance hemodialysis patients.

**Materials and methods:**

One hundred twenty-nine maintenance hemodialysis patients who had successful parathyroidectomy during 2012–2018 were identified (PTX group) and matched 1:1 to 479 patients with parathyroid hormone (PTH) levels ≤1,000 pg./mL (non-PTX control group) and 187 patients with PTH levels >1,000 pg./mL (pre-PTX control group) by propensity score. The matchings yielded 120 matched pairs from PTX and non-PTX groups (cohort 1) and 76 matched pairs from PTX and pre-PTX groups (cohort 2). Baseline and follow-up nutritional parameters associated with PEW were compared over the 12-month study period.

**Results:**

In cohort 1, substantially lower serum albumin and serum creatinine/body surface area (Cr/BSA) and higher proportions of patients with serum albumin ≤38 g/L (low albumin) and serum Cr/BSA ≤380 μmol/L/m^2^ (low Cr/BSA) were observed in the PTX group. These parameters improved significantly after parathyroidectomy. Total lymphocyte count (TLC) was comparable at baseline but the percentage of patients with TLC <800 cells/mm^3^ (low TLC) decreased substantially after parathyroidectomy. At follow-up, serum albumin, serum Cr/BSA and proportions of patients with low albumin and Cr/BSA became comparable with the non-PTX control group. The percentage of patients with low TLC became lower in the PTX group. Mixed-models analysis confirmed significant differences in the changes in serum albumin, serum Cr/BSA, and proportions of patients with low albumin and TLC between the two groups. In cohort 2, nutritional parameters were comparable at baseline. At follow-up, serum Cr/BSA was higher and proportions of patients with body mass index ≤18.5 kg/m^2^, low TLC and low Cr/BSA were lower in the PTX group. Weight gain was more frequent and of greater magnitude in the PTX group in both cohorts. A substantial reduction in blood pressure was also observed in the PTX group.

**Conclusion:**

Severe hyperparathyroidism was associated with nutritional impairment which improved considerably after parathyroidectomy.

## Introduction

Parathyroidectomy continues to play an important role in the treatment of severe hyperparathyroidism in patients with end-stage kidney disease (ESKD) especially in countries where accessibility to calcimimetics is limited. In addition to increasing bone mass and reducing fracture risk, improvements in quality of life, cardiovascular and overall survival have also been reported in several large observational studies from different parts of the world (1–4). The benefits of parathyroidectomy are still far reaching and likely extend beyond the mineral outcomes.

Protein-energy wasting (PEW) is prevalent in ESKD and is a powerful predictor of outcomes. Multiple mechanisms are involved including inadequate intake, inflammation, acidosis, hormonal imbalance, and dialysis procedure that lead to protein and fat breakdown (5). Until recently, hyperparathyroidism has never really been linked to malnutrition. The large epidemiological data of 42,319 maintenance hemodialysis (HD) patients revealed an association between increasing parathyroid hormone (PTH) level and 12-month weight loss (6). The recent investigation by our group also revealed worsening nutritional impairment among maintenance HD patients with severe hyperparathyroidism despite stable dietary protein intake (7). Other small studies have reported an increase in body weight, muscle strength and serum albumin after parathyroidectomy (8–11). These findings suggested adverse impacts of hyperparathyroidism on nutritional status and parathyroidectomy might convey favorable nutritional outcome. The previous studies on parathyroidectomy and nutrition were small, uncontrolled, and nutritional parameters were not systemically evaluated making it difficult to draw any meaningful conclusion.

The aim of the present study was to explore a relationship between parathyroidectomy and improvement in nutritional status in patients receiving maintenance HD. The changes in nutritional parameters associated with PEW in parathyroidectomized patients were compared with two non-parathyroidectomized control groups. The first control group represented HD patients whose PTH levels were in the recommended range or mildly elevated. The second control group consisted of patients with a more severe degree of hyperparathyroidism or awaiting parathyroidectomy.

## Materials and methods

### Study design and setting

The present study is a propensity-score matched retrospective cohort study of patients receiving maintenance HD at Ramathibodi hospital, Mahidol university, Bangkok, Thailand. The study was approved by the Human Research Ethics Committee of Faculty of Medicine, Ramathibodi Hospital, Mahidol University (approval numbers MURA2021/488, MURA2019/59 and MURA2017/220). Written informed consents were waived. The study was performed in accordance with the ethical standards laid down in the 1964 Declaration of Helsinki and its later amendments.

### Participants

**Figure 1:**
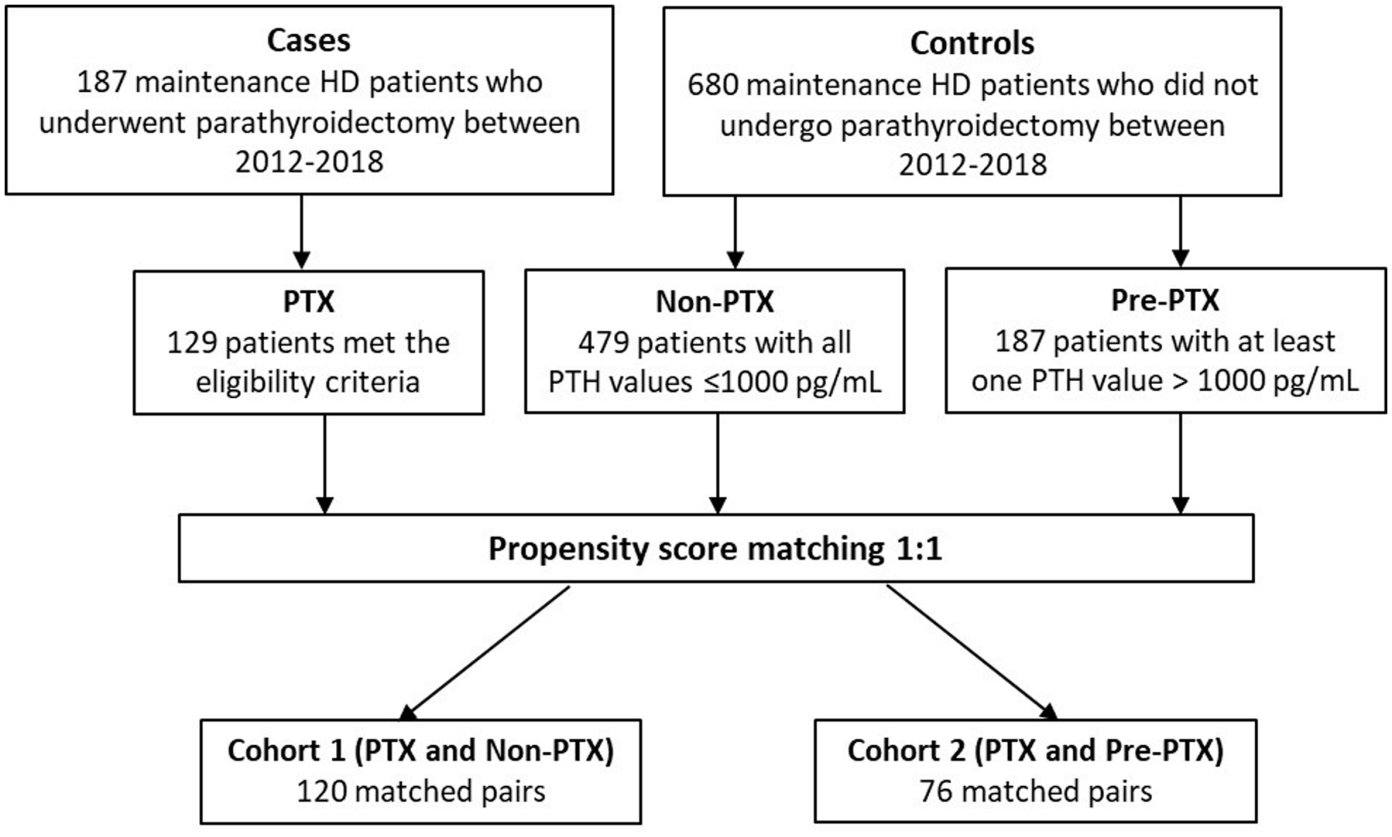
Study flow chart PTX, parathyroidectomy; PTH, parathyroid hormone.

The selection process of cases and controls are illustrated in Figure 1. All patients with ESKD receiving maintenance HD between 2012–2018 who underwent parathyroidectomy were identified through the hospital database. The eligibility criteria were: (1) availability of biochemical data 6 months prior to and 3–12 months after parathyroidectomy; and (2) reduction in PTH level > 50% at 6 weeks or > 25% at 12 months after parathyroidectomy. A total of 187 patients were identified and 129 patients met the eligibility criteria (PTX group). Among the eligible patients, 66.4% underwent subtotal parathyroidectomy, 25.8% underwent total parathyroidectomy with implantation and 7.8% underwent total parathyroidectomy without implantation. At discharge, 93.7% had PTH level < 585 pg./mL (9 times the upper normal limit) and 74% had PTH level < 130 pg./mL (2 times the upper normal limit). Minor operative complications including bleeding, infection and thrombosis of the AV access occurred in 9.4% and cardiovascular events occurred in 3.4% of the patients. The 30-day and 90-day mortality rates were 0 and 1.6%, respectively.

The two control groups consisted of HD patients who did not undergo parathyroidectomy during the study period. They had to have at least 4 sets of laboratory and nutritional data available for the consecutive 12-month period. Six hundred eighty non-parathyroidectomized patients were identified and 666 patients met these criteria. The first control group included patients with PTH levels within the target range or mild hyperparathyroidism (non-parathyroidectomy or non-PTX group). To be eligible for the non-PTX control group, all PTH values during the 12-month period had to be ≤1,000 pg./mL. The second control group consisted of patients with a more severe degree of hyperparathyroidism or those on the waiting list for parathyroidectomy (pre-parathyroidectomy or pre-PTX group). To be eligible for the pre-PTX control group, at least one PTH value during the 12-month period had to be >1,000 pg./mL. The cut-off PTH levels used in this study were adapted from the findings from our previous published study that showed no differences in nutritional parameters between the group of patients with PTH between 200–599 pg./mL and 600–1,500 pg./mL, whereas substantial deterioration of nutritional status was observed in the group of patients with PTH >1,500 pg./mL (7). Due to limited number of patients with extreme PTH levels (>1,500 pg./mL) that had not undergone parathyroidectomy for as long as 12 months, the cut-off value at 1000 pg./mL was selected in order to attain sufficient number of patients for the two non-parathyroidectomized control groups that represented different degree of hyperparathyroidism. Four hundred seventy-nine and 187 patients met the eligibility criteria for non-PTX and pre-PTX control groups, respectively.

To minimize the differences in baseline demographic data, 1:1 propensity score matchings were performed between PTX and non-PTX groups and between PTX and pre-PTX groups according to age, diabetes mellitus and dialysis vintage. These factors were chosen because they were important predictors of nutritional status and the degree of hyperparathyroidism in dialysis patients (12, 13). These yielded 120 matched pairs from PTX and non-PTX groups (cohort 1) and 76 matched pairs from PTX and pre-PTX groups (cohort 2). All patients received a 4-h, twice or trice weekly conventional HD session using high flux polysulfone dialyzer with a surface area between 1.8–2.1 m^2^. The dialysate sodium concentration ranged between 136–140 mEq/L, potassium 2–3 mEq/L, calcium 2.5–3.5 mEq/L, magnesium 0.7 mEq/L and bicarbonate 32–36 mEq/L.

### Outcomes

Primary outcomes were differences in nutritional parameters at baseline and follow-up between the parathyroidectomy and the two control groups. Secondary outcomes were between group differences in the changes of nutritional parameters from baseline and factors associated with nutritional impairment.

### Biochemical data

Demographic and laboratory data were obtained from the electronic medical record system. Body weight, height and blood pressure were obtained from the records of outpatient clinic visit on non-dialysis days. To qualify for using phosphate binders (calcium carbonate, calcium acetate, lanthanum carbonate and sevelamer carbonate), active vitamin D (calcitriol and alfacalcidol) or calcimimetics (cinacalcet), the medications had to be prescribed for at least two consecutive months. To ensure that calcium was prescribed for phosphate binding, the dose of elemental calcium had to be at least 1,000 mg/day and the frequency at least twice daily. To ensure that active vitamin D was prescribed for lowering PTH, the dose had to be at least 1.75 μg/week for calcitriol or 3 μg/week for alfacalcidol. Neutrophil-to-lymphocyte ratio (NLR) was selected as a marker of inflammation because the data were available in all patients (14). NLR ≥3.5 has been shown to predict significant inflammation and mortality in patients receiving maintenance HD (15, 16). Serum calcium was corrected based on the following equation: corrected calcium (mg/dl) = serum calcium (mg/dl) + [(40 – serum albumin (g/l))/10 × 0.8]. For PTX group, the baseline biochemical data were six-month average values prior to the date of parathyroidectomy and the follow-up biochemical data were average values between 3–9 months after the date of parathyroidectomy. The biochemical data during the first 3 months after parathyroidectomy were excluded because this was the period of recuperation and recovery from surgery. The rapid decline in PTH and fluctuations in serum calcium and phosphate from hungry bone syndrome were common during this period. For non-PTX and pre-PTX control groups, the biochemical data from the 12-month period preceding the last available lab for each patient were used. This 12-month period was divided into two 6-month blocks and the averages for each block were calculated. The average values for the first 6-month period were used as baseline and the last 6-month period were used as follow-up. Blood pressure, body weight and laboratory data obtained during acute illnesses were excluded.

### Nutritional assessment

The criteria for PEW proposed by the International Society of Renal Nutrition and Metabolism were used in the assessment of nutritional status. Serum albumin represented serum biochemistry, body mass index (BMI) represented body mass, serum creatinine/body surface area (Cr/BSA) represented skeletal muscle mass, and normalized protein catabolic rate (nPCR) represented protein intake. The cut-off values for serum albumin and serum Cr/BSA were 38 g/L and 380 μmol/L/m^2^, respectively (17, 18). The cut-off value for BMI at ≤18.5 kg/m^2^ was selected because of the small body size of Thai people and its association with an increase in mortality in Japanese HD patients (19, 20). BSA was calculated using DuBois & DuBois equation: 0.20247 x height (m)^0.725^ x weight (kg)^0.425^. Thailand Renal Replacement Therapy Registry provided the data on nPCR and adequacy of hemodialysis (Kt/V). Electronic records of HD treatment were available from 2016 onward and the data on Kt/V and nPCR were available in 77 and 40 patients in cohort 1 and cohort 2, respectively. Total lymphocyte count (TLC) was also included as another mean of nutritional assessment. The cut-off value for lymphopenia at ≤800 cells/mm^3^ was selected based on its association with an increase in mortality in HD patients (21). Serum cholesterol was excluded due to the interference by lipid lowering medications.

### Statistical methods

Data are presented as mean ± standard deviation, median (interquartile range) or mean (95% confidence interval). Propensity score matching was performed with a matching tolerance of 0.05, without replacement, and by giving priority to exact matches. The differences between the two groups were compared using Student’s T-test, Mann–Whitney U test or Chi-square test. Linear fixed and mixed-effects regression models were used to analyze the differences between baseline and follow-up biochemical data within the same group and between two groups over the study period. Linear regression analysis was used to analyze factors associated with an increase in the BMI. The relationships between two continuous variables were evaluated by Pearson correlation. *p*-value <0.05 was considered statistically significant. All statistical analyses were performed using the IBM SPSS Statistics for Windows, version 26 (IBM Corp., Armonk, N.Y., United States).

## Results

**Figure 2:**
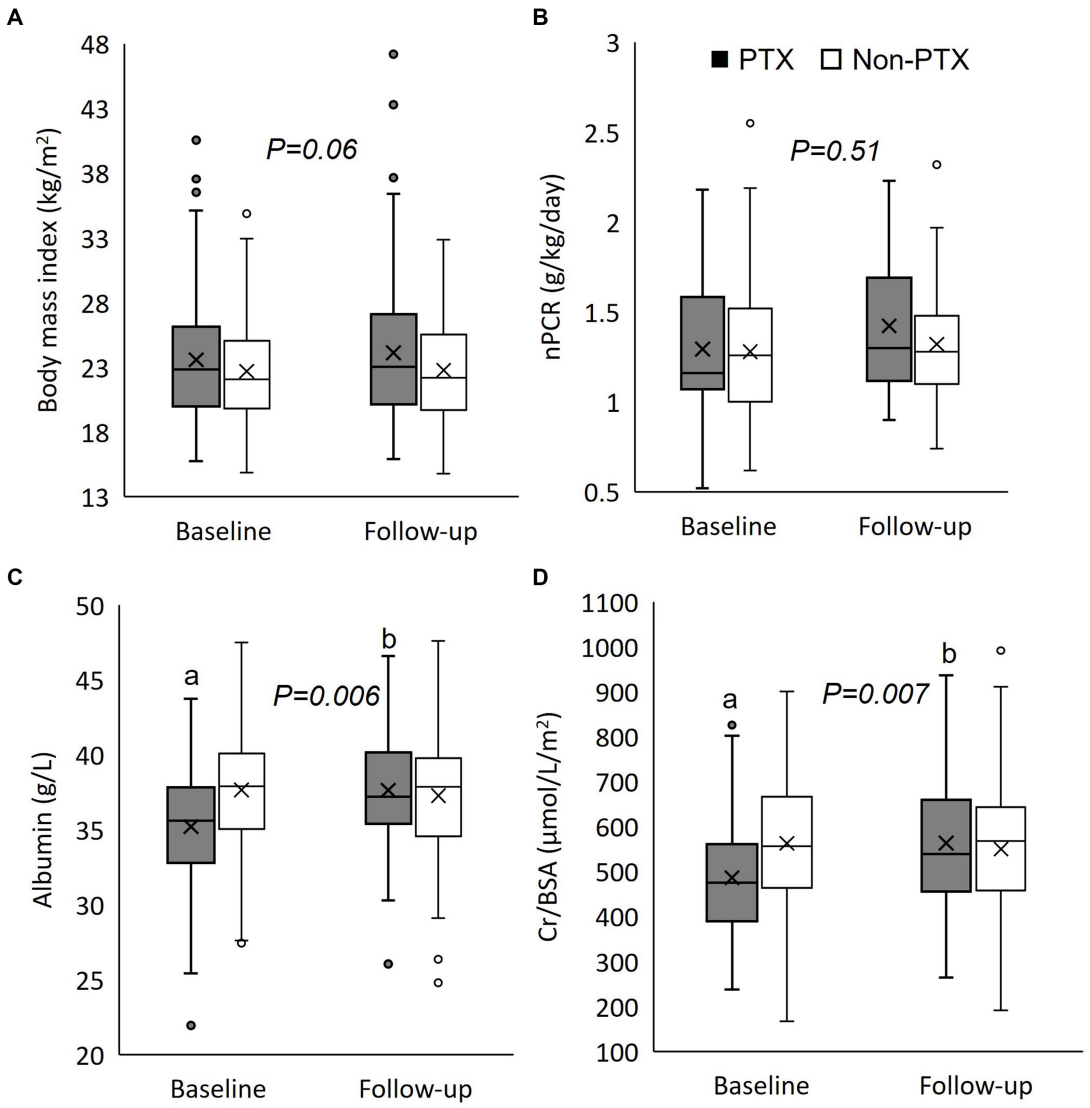
Box plot graphs illustrating the changes in nutritional parameters between parathyroidectomy and non-parathyroidectomy groups (cohort 1). **(A)** body mass index; **(B)** normalized protein catabolic rate; **(C)** serum albumin; and **(D)** serum creatinine/body surface area. PTX, parathyroidectomy; nPCR, normalized protein catabolic rate; Cr/BSA, creatinine/body surface area. The box represents interquartile range. The horizontal line and the X mark within the box represent median and mean, respectively. The whiskers represent 95% confidence interval. ^a^*p < 0.001* compared with non-parathyroidectomy group of the same period; ^b^*p < 0.001* compared with baseline value of the same group. The *p*-value in the graph represents the significance of between group changes over the study period.

**Figure 3:**
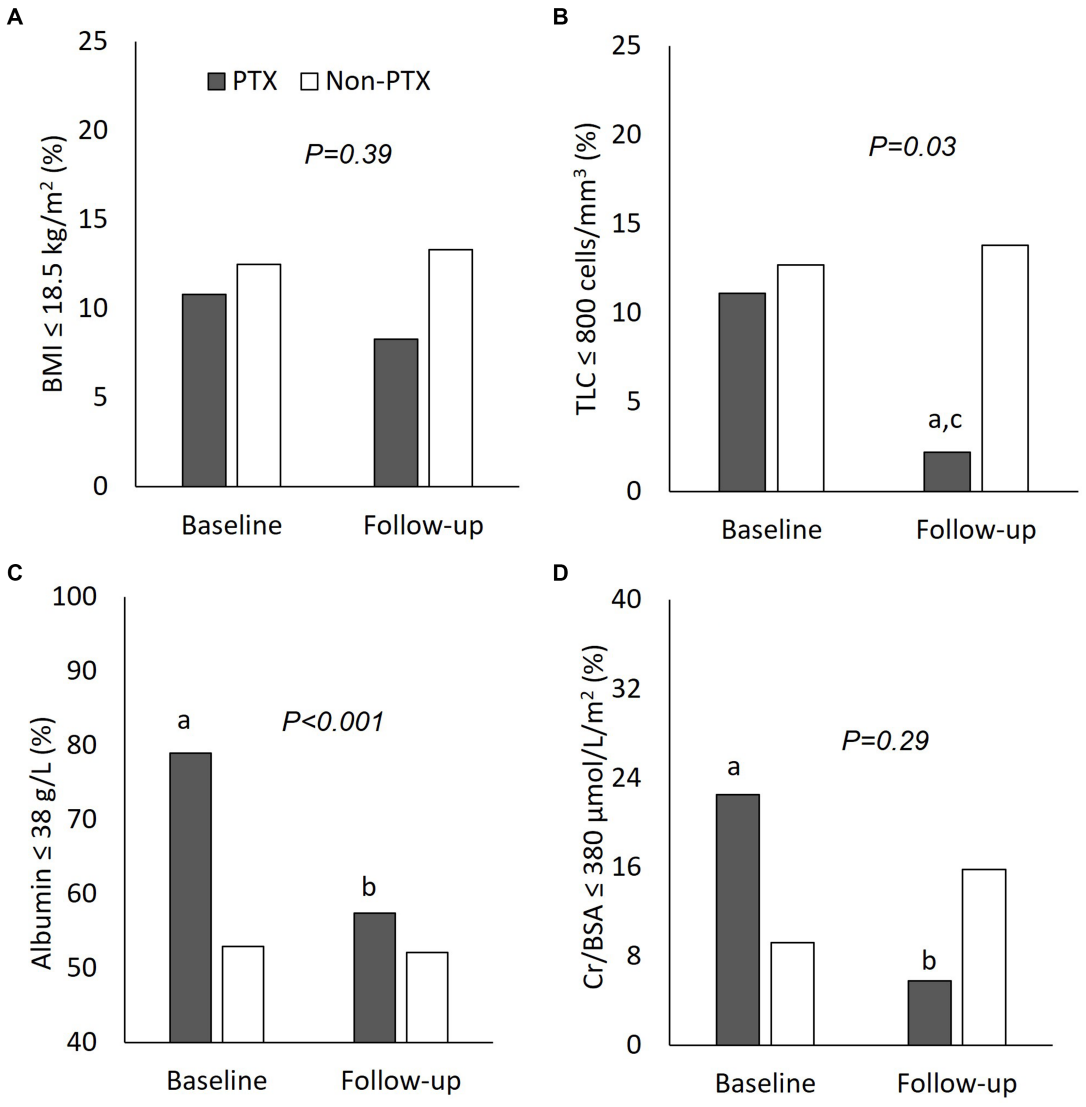
Comparisons of nutritional parameters between parathyroidectomy and non-parathyroidectomy groups (cohort 1). **(A)** body mass index ≤18.5 g/m^2^; **(B)** total lymphocyte count ≤800 cells/mm^3^; **(C)** serum albumin ≤38 g/L; and **(D)** serum creatinine/body surface area ≤ 380 μmol/L/m^2^. PTX, parathyroidectomy; BMI, body mass index; TLC, total lymphocyte count; Cr/BSA, creatinine/body surface area. ^a^*P < 0.01* compared with non-parathyroidectomy group of the same period; ^b^*P < 0.01 and ^c^P < 0.05* compared with baseline value of the same group. The *p*-value in the graph represents the significance of between group changes over the study period.

Figure 1 illustrates the number of participants at each stage of the study. One hundred-twenty matched pairs of patients from PTX and non-PTX groups (cohort 1) and seventy-six matched pairs of patients from PTX and pre-PTX groups (cohort 2) were included in the final analysis. Baseline demographics of the patients in both cohorts were mostly comparable except for lower proportions of patients in the non-PTX group were prescribed phosphate binders and calcimimetics (Table 1). Comparisons of blood pressure, laboratory data and nutritional parameters of the patients in cohort 1 at baseline and follow-up are shown in Table 2. At baseline, the two groups were equivalent in blood pressure, NLR, serum phosphate, Kt/V, BMI, TLC, nPCR and proportions of patients with BMI ≤18.5 kg/m^2^ and TLC ≤800 cells/mm^3^. Hemoglobin, serum albumin and Cr/BSA were lower, whereas proportions of patients with serum albumin ≤38 g/L and Cr/BSA ≤380 μmol/L/m^2^ were higher in the PTX group. At follow-up, systolic and pulse pressure were lower in the PTX group. Serum albumin, Cr/BSA and proportions of patients with serum albumin ≤38 g/L and Cr/BSA ≤380 μmol/L/m^2^ became equivalent to the non-PTX control group. Moreover, hemoglobin and BMI became higher and the percentage of patients with TLC ≤800 cells/mm^3^ became lower in the PTX group at follow-up. Table 3; Figures 2, 3 showed the results of mixed-effects regression analyses of patients in cohort 1. After parathyroidectomy, a significant decline in systolic, diastolic and pulse pressure was observed. Hemoglobin, albumin, and Cr/BSA increased and proportions of patients with TLC ≤800 cells/mm^3^, serum albumin ≤38 g/L and Cr/BSA ≤380 μmol/L/m^2^ decreased substantially after parathyroidectomy. These parameters were largely unchanged in the non-PTX control group. There were no changes in the NLR, BMI, nPCR and proportion of patients with BMI ≤18.5 kg/m^2^ in either group. Between group comparisons confirmed significant differences in the alterations in serum albumin, Cr/BSA, and proportions of patients with TLC ≤800 cells/mm^3^ and serum albumin ≤38 g/L during the study period.

**Table 1:** Baseline demographics of all patients.

Parameters	Cohort 1			Cohort 2		
	PTX *N* = 120	Non-PTX *N* = 120	*p*	PTX *N* = 76	Pre-PTX *N* = 76	*p*
Age (yrs)	46.43 ± 13.67	45.13 ± 12.48	0.44	48.35 ± 14.17	51.28 ± 16.49	0.24
Male (*n*/%)	54 (45)	57 (47.5)	0.7	34 (44.7)	37 (48.7)	0.63
Body weight (kg)	59.5 ± 14.7	59.26 ± 12.8	0.9	60.13 ± 14.83	62.74 ± 16.14	0.3
Diabetes (*n*/%)	15 (12.5)	9 (7.5)	0.2	10 (13.2)	14 (18.4)	0.37
CVD (*n*/%)	16 (13.3)	11 (9.2)	0.31	13 (17.1)	18 (23.7)	0.3
Hypertension (*n*/%)	108 (90)	102 (85)	0.24	71 (93.4)	66 (86.8)	0.17
DV (months)	82.11 ± 42.29	71.87 ± 47.14	0.08	80.55 ± 46.59	84.61 ± 50.21	0.61
Twice/week HD (*n*/%)	15 (12.5)	20 (16.7)	0.36	11 (14.5)	16 (21.3)	0.27
Arteriovenous access			0.96			0.76
Fistula (*n*/%)	85 (70.8)	86 (72.3)		52 (68.4)	49 (64.5)	
Graft (*n*/%)	24 (20)	22 (18.5)		16 (21.1)	16 (21.1)	
Catheter (*n*/%)	11 (9.2)	11 (9.2)		8 (10.5)	11 (14.5)	
Phosphate Binders			0.002			0.26
None (*n*/%)	14 (11.7)	38 (31.7)		10 (13.2)	9 (11.8)	
Calcium (*n*/%)	99 (82.5)	76 (63.3)		59 (77.6)	61 (80.3)	
Non-Calcium (*n*/%)	24 (20)	21 (17.5)		18 (23.7)	27 (35.5)	
Combined (*n*/%)	15 (12.5)	15 (12.5)		11 (14.5)	21 (27.6)	
Active vitamin D (*n*/%)	79 (65.8)	70 (58.3)	0.23	55 (72.4)	59 (77.6)	0.45
Calcimimetics (*n*/%)	37 (30.8)	12 (10)	<0.001	22 (28.9)	23 (30.3)	0.86

PTX, parathyroidectomy; PTH, parathyroid hormone; CVD, cardiovascular disease; HD, hemodialysis.

**Table 2:** Comparisons of blood pressure, laboratory and nutritional parameters between parathyroidectomy and non-parathyroidectomy groups at baseline and follow-up (cohort 1).

Parameters	Baseline			Follow-up		
	PTX	Non-PTX	*p*	PTX	Non-PTX	*p*
Blood Pressure (mmHg)						
Systolic	145.7 ± 21.57	141.9 ± 19.91	0.16	134.7 ± 23.1	140.8 ± 19.64	0.03
Diastolic	83.26 ± 11.79	82.06 ± 10.46	0.42	79.46 ± 12.24	81.51 ± 10.55	0.18
Pulse Pressure	62.61 ± 17.03	60 ± 14.98	0.22	55.31 ± 16.8	59.43 ± 14.32	0.047
Laboratory data						
Hemoglobin (g/dL)	10.36 ± 1.84	10.85 ± 1.83	0.04	11.7 ± 1.95	10.75 ± 1.71	<0.001
NLR	3.67 ± 1.7	3.71 ± 1.7	0.86	3.52 ± 1.43	3.68 ± 1.64	0.48
NLR ≥3.5 (*n*/%)	48 (40)	44 (36.7)	0.5	39 (32.5)	43 (35.8)	0.58
Calcium (mg/dL)	10.16 ± 0.84	9.73 ± 0.96	<0.001	8.88 ± 1.94	9.82 ± 0.88	<0.001
Phosphate (mg/dL)	5.22 ± 1.63	5.13 ± 1.46	0.65	3.86 ± 1.52	5.22 ± 1.52	<0.001
PTH (pg/mL)	2,205 (1574–3,096)	374 (229–546)	<0.001	68 (18–281)	388 (253–706)	<0.001
Kt/V	1.86 ± 0.51	1.84 ± 0.35	0.85	1.94 ± 0.56	1.92 ± 0.4	0.91
Nutritional data						
BMI (kg/m^2^)	23.58 ± 4.86	22.7 ± 3.99	0.13	24.13 ± 5.3	22.77 ± 4.08	0.03
TLC (cells/mm^3^)	1,361 ± 461	1,349 ± 464	0.84	1,471 ± 470	1,424 ± 631	0.56
nPCR (g/kg/day)	1.29 ± 0.4	1.28 ± 0.37	0.89	1.42 ± 0.41	1.32 ± 0.33	0.3
Albumin (g/L)	35.21 ± 3.91	37.67 ± 3.71	<0.001	37.6 ± 3.63	37.28 ± 3.81	0.48
Cr/BSA (μmol/L/m^2^)	487 ± 129	563 ± 150	<0.001	563 ± 145	550 ± 152	0.55
BMI ≤ 18.5 (*n*/%)	13 (10.8)	15 (12.5)	0.67	10 (8.3)	16 (13.3)	0.22
TLC ≤ 800 (*n*/%)	12 (11.1)	14 (12.7)	0.71	2 (2.2)	15 (13.8)	0.004
Albumin ≤38 (*n*/%)	94 (79)	63 (52.9)	<0.001	58 (57.4)	61 (52.1)	0.43
Cr/BSA ≤ 380 (*n*/%)	27 (22.5)	11 (9.2)	0.007	7 (5.8)	19 (15.8)	0.06

Data are presented as mean ± standard deviation or median (interquartile range); PTX, parathyroidectomy; NLR, neutrophil-to-lymphocyte ratio; PTH, parathyroid hormone; BMI, body mass index; TLC, total lymphocyte count; nPCR, normalized protein catabolic rate; Cr/BSA, body surface area; creatinine/body surface area.

**Table 3:** Mixed-effects regression analyses of the changes in blood pressure, laboratory data and nutritional parameters from baseline between parathyroidectomy and non-parathyroidectomy groups (cohort 1).

Parameters	Time	Mean (95% confidence interval)		Within group comparisons (Mean change from baseline)				Between group comparisons (PTX vs. Non-PTX)	*P*
		PTX	Non-PTX	PTX	*P*	Non-PTX	*P*		
Systolic BP (mmHg)	Baseline	145.7 (141.6,149.9)	141.9 (138.3,145.5)	-11 (-16.9,-5.18)	<0.001	-1.1 (-6.2,4)	0.67	-1.54 (-6.37,3.29)	0.53
	Follow-up	134.7 (130.6,138.8)	140.8 (137.2,144.5)						
Diastolic BP (mmHg)	Baseline	83.26 (81.03,85.49)	82.06 (80.15,83.96)	-3.8 (-6.93,-0.66)	0.02	-0.55 (-3.26,2.17)	0.69	-0.67 (-3.32,1.98)	0.62
	Follow-up	79.46 (77.25,81.67)	81.51 (79.58,83.44)						
Pulse Pressure (mmHg)	Baseline	62.61 (59.48,65.75)	60 (57.34,62.66)	-7.3 (-11.7,-2.88)	0.001	-0.57 (-4.36,3.21)	0.77	-0.96 (-4.63,2.71)	0.61
	Follow-up	55.31 (52.21,58.42)	59.43 (56.73,62.12)						
Hemoglobin (g/dL)	Baseline	10.36 (10.02,10.7)	10.85 (10.53,11.17)	1.34 (0.82,1.86)	<0.001	-0.09 (-0.55,0.36)	0.68	0.06 (-0.36,0.49)	0.77
	Follow-up	11.7 (11.3,12.1)	10.75 (10.43,11.08)						
NLR	Baseline	3.67 (3.37,3.97)	3.71 (3.39,4.03)	-0.15 (-0.59,0.3)	0.51	-0.03 (-0.48,0.41)	0.89	-0.08 (-0.47,0.32)	0.71
	Follow-up	3.52 (3.19,3.85)	3.68 (3.36,3.99)						
NLR ≥3.5 (n/%)	Baseline	48 (40)	44 (36.7)	-0.02 (-0.16,0.13)	0.83	-0.01 (-0.14,0.12)	0.89	0.05 (-0.07,0.16)	0.4
	Follow-up	39 (32.5)	43 (35.8)						
TLC (cells/mm^3^)	Baseline	1361 (1273,1449)	1349 (1245,1453)	109 (-21.5,240)	0.1	75.4 (-72.1,223)	0.32	14.81 (-106,136)	0.81
	Follow-up	1471 (1374,1567)	1424 (1319,1528)						
BMI (kg/m^2^)	Baseline	23.58 (22.67,24.49)	22.7 (21.97,23.43)	0.55 (-0.76,1.85)	0.41	0.07 (-0.97,1.12)	0.89	1.11 (-0.05,2.27)	0.06
	Follow-up	24.13 (23.2,25.06)	22.77 (22.03,23.52)						
nPCR (g/kg/day)	Baseline	1.29 (1.09,1.5)	1.28 (1.19,1.37)	0.13 (-0.16,0.42)	0.37	0.04 (-0.08,0.17)	0.5	0.06 (-0.12,0.23)	0.51
	Follow-up	1.42 (1.22,1.63)	1.32 (1.23,1.41)						
Albumin (g/L)	Baseline	35.21 (34.53,35.89)	37.67 (36.99,38.35)	2.44 (1.43,3.44)	<0.001	-0.39 (-1.35,0.58)	0.43	-1.23 (-2.1,-0.36)	0.006
	Follow-up	37.62 (36.9,38.38)	37.28 (36.59,37.96)						
Cr/BSA (μmol/L/m^2^)	Baseline	487 (462,511)	563 (535,591)	76.5 (38.4,115)	<0.001	-12.9 (-53.2,27.2)	0.53	-47.3 (-81.8,-12.9)	0.007
	Follow-up	563 (534,593)	550 (521,579)						
BMI ≤18.5 (n/%)	Baseline	13 (10.8)	15 (12.5)	-0.02 (-0.1,0.05)	0.57	0.01 (-0.08,0.1)	0.81	-0.03 (-0.11,0.04)	0.39
	Follow-up	10 (8.3)	16 (13.3)						
TLC ≤800 (n/%)	Baseline	12 (11.1)	14 (12.7)	-0.09 (-0.16,-0.02)	0.02	0.1 (-0.08,0.1)	0.82	-0.08 (-0.15,-0.01)	0.03
	Follow-up	2 (2.2)	15 (13.8)						
Albumin ≤38 (n/%)	Baseline	94 (79)	63 (52.9)	-0.22 (-0.34,-0.09)	0.001	-0.008 (-0.14,0.12)	0.9	0.19 (0.09,0.3)	<0.001
	Follow-up	58 (57.4)	61 (52.1)						
Cr/BSA ≤380 (n/%)	Baseline	27 (22.5)	11 (9.2)	-0.14 (-0.25,-0.14)	0.007	0.08 (-0.01,0.17)	0.09	0.04 (-0.04,0.12)	0.29
	Follow-up	7 (5.8)	19 (15.8)						

Data are presented as mean (95% confidence interval). PTX, parathyroidectomy; BP, blood pressure; NLR, neutrophil-to-lymphocyte ratio; BMI, body mass index; TLC, total lymphocyte count; Cr/BSA, serum creatinine/body surface area.

**Figure 4:**
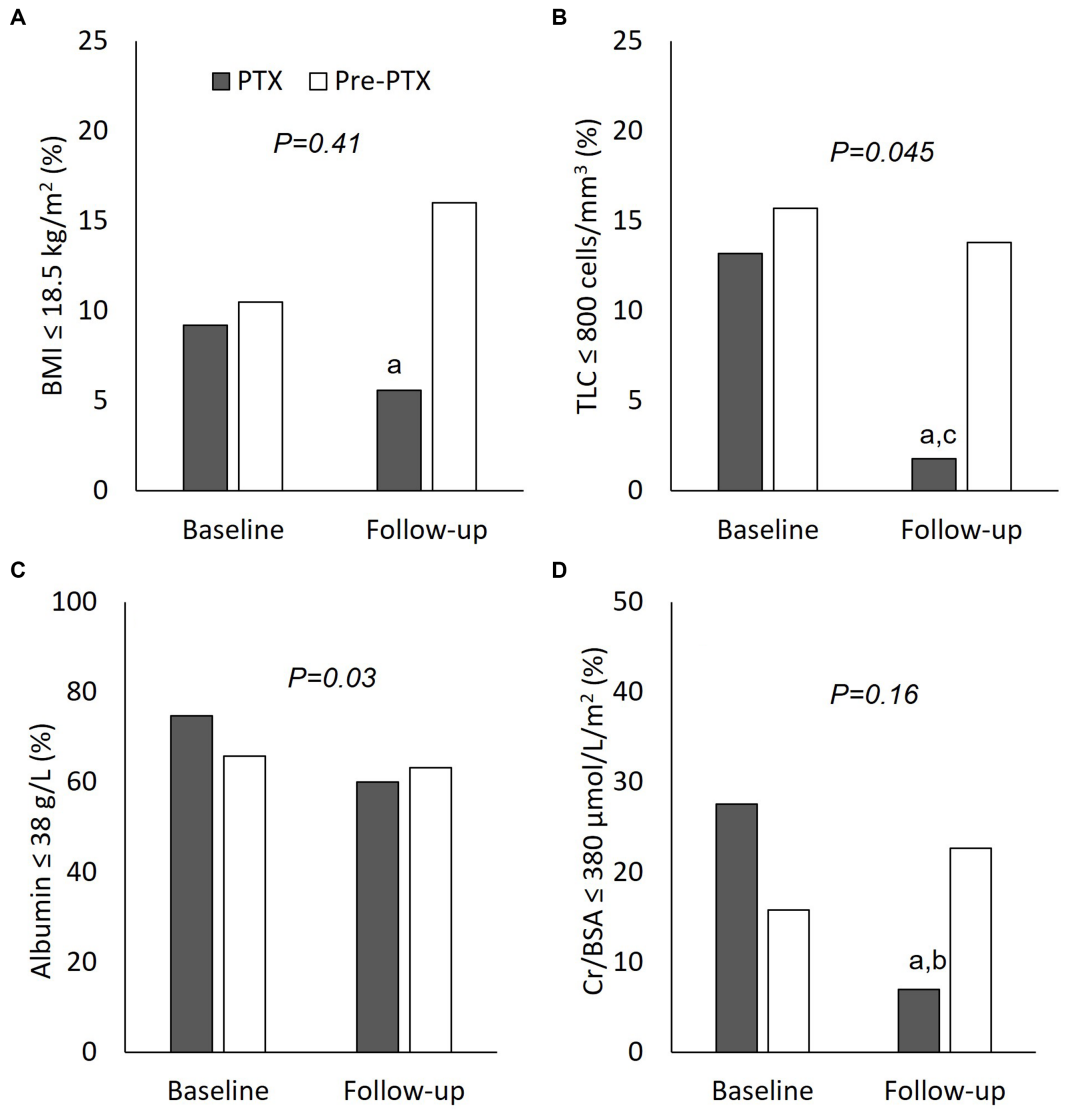
Comparisons of nutritional parameters between parathyroidectomy and pre-parathyroidectomy groups (cohort 2). **(A)** body mass index ≤18.5 g/m^2^; **(B)** total lymphocyte count ≤800 cells/mm^3^; **(C)** serum albumin ≤38 g/L; and **(D)** serum creatinine/body surface area ≤ 380 μmol/L/m^2^. PTX, parathyroidectomy; BMI, body mass index; TLC, total lymphocyte count; Cr/BSA, creatinine/body surface area. ^a^*P < 0.05* compared with pre-parathyroidectomy group of the same period; ^b^*P < 0.01 and ^c^P < 0.05* compared with baseline value of the same group The *p*-value in the graph represents the significance of between group changes over the study period.

The biochemical data of patients in cohort 2 at baseline and follow-up are shown in Tables 4, 5; Figure 4. Patients in the pre-PTX group had a less severe degree of hyperparathyroidism compared with patients in the PTX group as indicated by lower PTH and serum calcium levels. This was inevitable because it was not possible to have a control group with similar degree of hyperparathyroidism that had not undergone parathyroidectomy for as long as 12 months. At baseline, blood pressure, other laboratory and nutritional data were equivalent among the two groups. At follow-up, systolic and pulse pressure were lower in the PTX group. Higher serum Cr/BSA and lower proportions of patients with BMI ≤18.5 kg/m^2^, TLC ≤800 cells/mm^3^ and Cr/BSA ≤380 μmol/L/m^2^ were observed in the PTX group. Within group comparisons showed similar findings for the PTX group as in cohort 1, whereas all parameters were largely unchanged in the pre-PTX group. Between group comparisons revealed significant differences in the alterations in pulse pressure and proportions of patients with TLC ≤800 cells/mm^3^ and serum albumin ≤38 g/L.

**Table 4:** Comparisons of laboratory and nutritional parameters between parathyroidectomy and pre-parathyroidectomy groups at baseline and follow-up (cohort 2).

Parameters	Baseline			Follow-up		
	PTX	Pre-PTX	*p*	PTX	Pre-PTX	*p*
Blood Pressure (mmHg)						
Systolic	144.9 ± 23.38	145.1 ± 19.02	0.95	134.4 ± 24.06	144.3 ± 20.77	0.008
Diastolic	82.55 ± 12.7	80.25 ± 10.71	0.24	77.93 ± 12.66	79.86 ± 11.11	0.32
Pulse Pressure	62.39 ± 17.56	64.90 ± 18.58	0.4	56.43 ± 17.13	64.4 ± 19.16	0.008
Laboratory data						
Hemoglobin (g/dL)	10.68 ± 1.78	10.94 ± 1.56	0.34	11.39 ± 1.71	10.96 ± 1.75	0.16
NLR	3.68 ± 1.8	3.45 ± 1.74	0.44	3.61 ± 1.52	3.38 ± 1.43	0.39
NLR ≥ 3.5 (*n*/%)	29 (43.3)	26 (37.1)	0.46	25 (43.9)	28 (40)	0.66
Calcium (mg/dL)	10.28 ± 0.82	9.96 ± 0.9	0.02	9.49 ± 1.46	9.62 ± 0.97	0.54
Phosphate (mg/dL)	5.01 ± 1.68	5.53 ± 1.51	0.05	4.1 ± 1.6	5.56 ± 1.26	<0.001
PTH (pg/mL)	1713 (1427–2,180)	1,133 (746–1,597)	<0.001	39.8 (15–239)	1,615 (1127–2026)	<0.001
Kt/V	1.84 ± 0.28	1.77 ± 0.39	0.56	1.99 ± 0.69	1.75 ± 0.35	0.32
Nutritional data						
BMI (kg/m^2^)	23.82 ± 4.75	24.16 ± 5.31	0.68	24.46 ± 5.29	23.88 ± 5.14	0.5
TLC (cells/mm^3^)	1,383 ± 512	1,440 ± 565	0.53	1,482 ± 449	1,367 ± 478	0.17
nPCR (g/kg/day)	1.36 ± 0.38	1.25 ± 0.37	0.51	1.42 ± 0.46	1.19 ± 0.3	0.2
Albumin (g/L)	36.1 ± 3.56	36.49 ± 3.75	0.51	37.42 ± 3.46	36.49 ± 3.92	0.14
Cr/BSA (μmol/L/m^2^)	487 ± 138	530 ± 150	0.07	551 ± 150	496 ± 141	0.03
BMI ≤ 18.5 (*n*/%)	7 (9.2)	8 (10.5)	0.79	4 (5.6)	12 (16)	0.04
TLC ≤ 800 (*n*/%)	9 (13.2)	11 (15.7)	0.38	1 (1.8)	10 (14.3)	0.01
Albumin ≤38 (*n*/%)	56 (74.7)	50 (65.8)	0.23	39 (60)	48 (63.2)	0.7
Cr/BSA ≤ 380 (*n*/%)	21 (27.6)	12 (15.8)	0.08	4 (7)	17 (22.7)	0.02

Data are presented as mean ± standard deviation or median (interquartile range); PTX, parathyroidectomy; NLR, neutrophil-to-lymphocyte ratio; PTH, parathyroid hormone; BMI, body mass index; TLC, total lymphocyte count; nPCR, normalized protein catabolic rate; Cr/BSA, creatinine/body surface area.

**Table 5:** Mixed-effects regression analyses of the changes in blood pressure and nutritional parameters from baseline between parathyroidectomy and pre-parathyroidectomy groups (cohort 2).

Parameters	Time	Mean (95% confidence interval)		Within group comparisons (Mean change from baseline)				Between group comparisons (PTX vs. Pre-PTX)	*P*
		PTX	Pre-PTX	PTX	*P*	Pre-PTX	*P*		
Systolic BP (mmHg)	Baseline	144.9 (139.4,150.5)	145.1 (140.6,149.7)	-10.6 (-18.4,-2.81)	0.008	-0.88 (-7.33,5.56)	0.79	-5.89 (-12.2,0.41)	0.07
	Follow-up	134.4 (128.9,139.8)	144.3 (139.7,148.8)						
Diastolic BP (mmHg)	Baseline	82.55 (79.58,85.52)	80.25 (77.76,82.74)	-4.62 (-8.77,-0.47)	0.03	-0.39 (-3.92,3.15)	0.83	-0.31 (-3.82,3.2)	0.86
	Follow-up	77.93 (75.04,80.82)	79.86 (77.36,82.37)						
Pulse Pressure (mmHg)	Baseline	62.39 (58.32,66.46)	64.9 (60.59,69.2)	-5.96 (-11.6,-0.28)	0.04	-0.5 (-6.61,5.61)	0.87	-5.74 (-11.1,-0.38)	0.04
	Follow-up	56.43 (52.47,60.39)	64.4 (60.06,68.73)						
BMI (kg/m^2^)	Baseline	23.82 (22.68,24.96)	24.16 (22.97,25.34)	0.64 (-0.99,2.28)	0.44	-0.28 (-1.96,1.4)	0.74	0.04 (-1.59,1.66)	0.97
	Follow-up	24.46 (23.3,25.63)	23.88 (22.69,25.07)						
TLC (cells/mm^3^)	Baseline	1383 (1267,1499)	1440 (1317,1564)	99.5 (-72.8,272)	0.26	-73.2 (-248,102)	0.41	16.5 (-142,175)	0.84
	Follow-up	1482 (1355,1609)	1367 (1244,1491)						
Albumin (g/L)	Baseline	36.1 (35.3,36.89)	36.49 (35.62,37.36)	1.32 (0.15,2.5)	0.03	-0.004 (-1.23,1.23)	0.99	0.19 (-0.92,1.29)	0.74
	Follow-up	37.42 (36.56,38.28)	36.49 (35.62,37.36)						
Cr/BSA (μmol/L/m^2^)	Baseline	487.7 (455.3,520.2)	530.2 (497.2,563.2)	63 (13.4,113)	0.01	-34.6 (-81.4,12.3)	0.15	-4.14 (-47.7,39.4)	0.85
	Follow-up	550.7 (513.2,588.3)	495.7 (462.5,528.9)						
BMI ≤18.5 (n/%)	Baseline	7 (9.2)	8 (10.5)	-0.04 (-0.12,0.05)	0.4	0.06 (-0.06,0.16)	0.32	0.02 (-0.02,0.06)	0.41
	Follow-up	4 (5.6)	12 (16)						
TLC ≤ 800 (n/%)	Baseline	9 (13.2)	11 (15.7)	-0.12 (-0.21,-0.02)	0.02	-0.01 (-0.13,0.11)	0.82	-0.06 (-0.11,-0.001)	0.045
	Follow-up	1 (1.8)	10 (14.3)						
Albumin ≤38 (n/%)	Baseline	56 (74.7)	50 (65.8)	-0.15 (-0.3,0.009)	0.07	-0.03 (-0.18,0.13)	0.74	-0.08 (-0.16,-0.007)	0.03
	Follow-up	39 (60)	48 (63.2)						
Cr/BSA ≤380 (n/%)	Baseline	21 (27.6)	12 (15.8)	-0.21 (-0.34,-0.07)	0.002	0.07 (-0.06,0.2)	0.29	-0.05 (-0.13,0.02)	0.16
	Follow-up	4 (7)	17 (22.7)						

Parameters from baseline between parathyroidectomy and non-parathyroidectomy groups (cohort 2).

Data are presented as mean (95% confidence interval). PTX, parathyroidectomy; BP, blood pressure; BMI, body mass index; TLC, total lymphocyte count; Cr/BSA, serum creatinine/body surface area.

**Figure 5:**
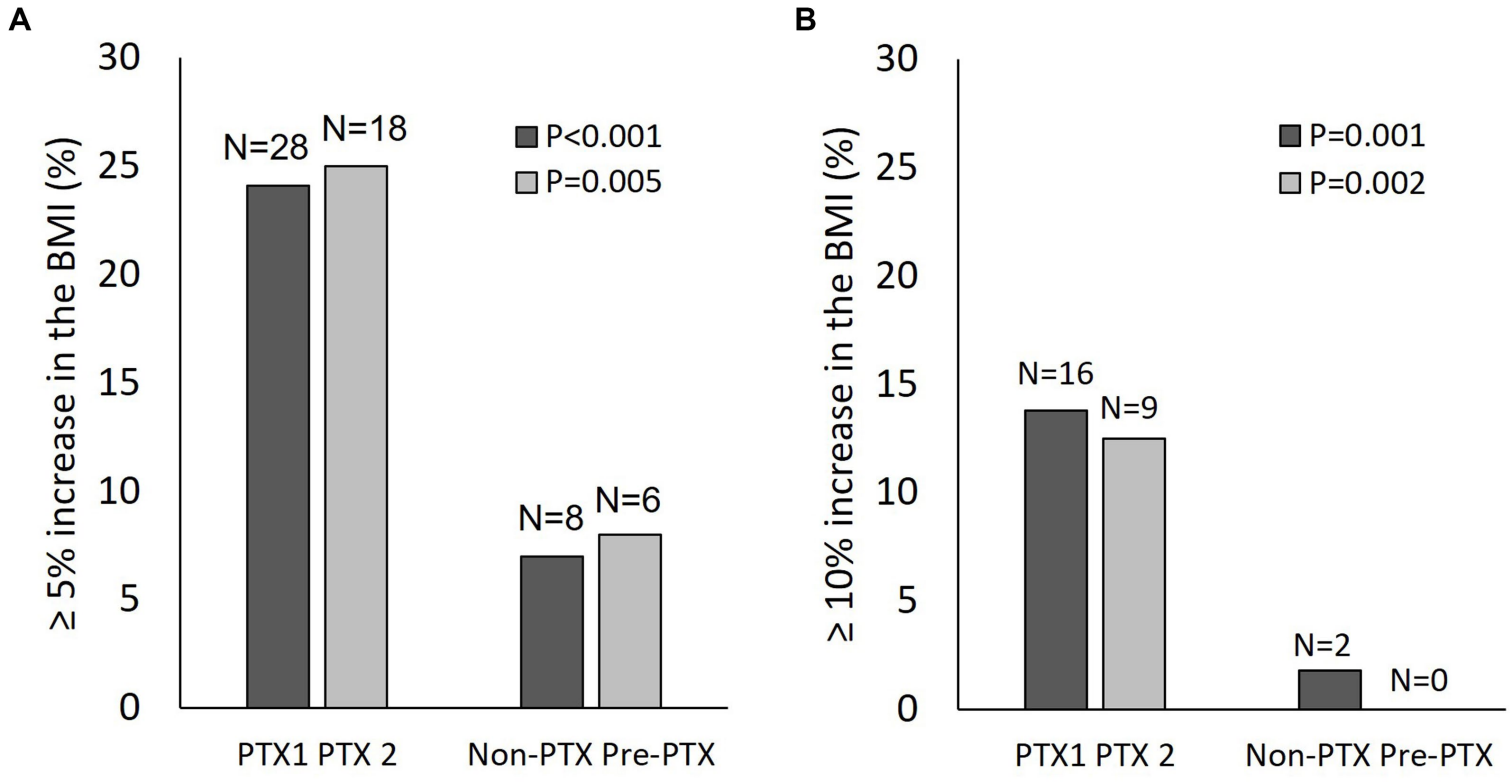
Changes in the body mass index in patients from cohort 1 and cohort 2. **(A)** proportion of patients with ≥5% increase in the body mass index from baseline; **(B)** proportion of patients with ≥10% increase in the body mass index from baseline. Black bars, cohort 1; grey bars, cohort 2; PTX, parathyroidectomy; PTX1, parathyroidectomy from cohort 1; PTX2, parathyroidectomy from cohort 2; BMI, body mass index.

Increases in the BMI ≥5% and ≥ 10% occurred more frequently in the PTX group compared with the non-PTX (24.1% vs. 7 and 13.8% vs.1.8%) and the pre-PTX (25% vs. 8 and 12.5% vs. 0%) control groups (Figure 5). Univariate linear regression analysis revealed negative associations between baseline serum albumin (beta (95% confidence interval) = −0.17 (−0.29,-0.04), *p* = 0.009) and Cr/BSA (−0.005 (−0.009,-0.001), *p* = 0.02) and positive associations between log PTH level (3.35 (1.33, 5.36), *p* = 0.001) and parathyroidectomy (2.86 (0.81,4.9), *p* = 0.006) with ≥10% increase in the BMI among patients in cohort 1. There were no relationships between age, sex, diabetes, baseline BMI, dialysis vintage, hemoglobin and NLR with ≥10% increase in the BMI (Supplementary Table S1). The changes in BMI also correlated positively with the changes in serum creatinine (r = 0.347, *p* < 0.001) and Cr/BSA (*r* = 0.247, *p* = 0.001) (Supplementary Figure S1). There were no correlations between the changes in BMI with the changes in hemoglobin, NLR, TLC and serum albumin. The alterations in TLC correlated positively with the alterations in hemoglobin (*r* = 0.151, *p* = 0.04) and creatinine (*r* = 0.165, *p* = 0.03). Similar findings were observed among patients in cohort 2 (data not shown).

## Discussion

This is the largest study with appropriate control groups that demonstrated an improvement in nutritional parameters after parathyroidectomy in patients receiving maintenance HD with severe hyperparathyroidism. The main findings included substantial nutritional impairment among patients with severe hyperparathyroidism, which improved considerably after parathyroidectomy. Several nutritional indices became equivalent to those with PTH levels in the recommended range or mild hyperparathyroidism and better than those with severe hyperparathyroidism. Weight gain was also more frequent and more substantial among patients who underwent parathyroidectomy. Mixed-model analyses confirmed significant differences in the changes of nutritional parameters between the PTX group and the two control groups. Incidentally, a reduction in blood pressure was also observed in the PTX group.

Patients with severe hyperparathyroidism had lower serum albumin and Cr/BSA compared with patients with PTH levels in the target range or mild hyperparathyroidism. Dietary intake as indicated by nPCR and inflammation as indicated by NLR were comparable between the two groups. These data suggested the presence of PEW and sarcopenia in patients with severe hyperparathyroidism that were unlikely to be due to decreased protein intake or inflammation. Inflammation and retention of uremic toxins are two main factors contributing to PEW in patients with kidney failure. The relationship between increased PTH level and inflammation has been somewhat inconsistent, however, in a large cohort of over 8,000 adults from United States, increased PTH level showed a positive correlation with the levels of inflammatory markers (22). Since causes of inflammation in ESKD are multifactorial and, even if the link between hyperparathyroidism and inflammation does exist, this can easily be overcome by other more powerful factors such as increased pro-inflammatory cytokines, oxidative stress, acidosis, infections, and intestinal dysbiosis (23).

Increased resting energy expenditure among patients with severe hyperparathyroidism compared with patients with mild to moderate hyperparathyroidism has been reported previously (24). Later studies in maintenance HD patients also demonstrated a relationship between increased serum calcium, phosphate and PTH with PEW (7, 25–27). The underlying mechanisms for this relationship have been explored in an animal model of kidney failure. The 5/6 nephrectomy mice with elevated PTH level experienced cachexia, reduced body weight and increased energy expenditure (28). The weight loss was associated with reduced fat and skeletal muscle mass that was not due to reduced food intake. The PTH gene over-expressed mice mimicking primary hyperparathyroidism showed adipose tissue browning leading to increased energy expenditure, reduced fat content, decreased body weight and muscle atrophy (29). Fat-specific knockout of PTH receptor in 5/6 nephrectomy mice was able to prevent adipose tissue browning, preserve and improve skeletal muscle mass and strength (28). These important findings explained the link between extreme PTH level and PEW in ESKD patients.

After parathyroidectomy, nutritional indices improved considerably. These findings supported the idea of hyperparathyroidism causing PEW and removal of excess PTH could improve nutritional status. Other small uncontrolled observational studies have reported similar observation (8–11, 30). Although the findings on the changes in the BMI were not consistent in the primary analysis, further analyses on the degree of the changes revealed a more frequent and a more substantial increase in the BMI in the PTX group compared with the two control groups. Moreover, higher baseline PTH level and lower baseline serum albumin and Cr/BSA, which were more common in the parathyroidectomy group, were significantly associated with ≥10% increase in the BMI. A fluctuation in extracellular fluid volume in HD patients could possibly interfere with the actual changes in body weight negating the positive results in the primary analysis. The recent large epidemiological data in patients receiving maintenance HD also showed an inverse relationship between PTH level and 12-month weight change (6). Moreover, the weight loss was more pronounced among patients with preserved appetites suggesting that the decrease in body weight was not the result of decreased dietary intake.

A considerable improvement in hematologic parameters including anemia and lymphopenia were observed after parathyroidectomy. Suppression of erythropoiesis in secondary hyperparathyroidism as a result of bone marrow fibrosis has been well documented (31). An adverse influence of hyperparathyroidism on the immune system has also been suggested (32). A reduction in the total number and the function of lymphocytes has been reported previously among ESKD patients with elevated PTH level (33, 34). Incidentally, the present study also found a reduction in systolic, diastolic and pulse pressure after parathyroidectomy. Other small observational studies have reported similar findings (35). The postulated mechanisms included correction of hypercalcemia, attenuation of vascular calcification and vascular stiffness and a direct effect of PTH on the vascular wall (36). Further clinical and experimental studies are needed to confirm the causality and to elucidate the mechanistic link between hyperparathyroidism and hypertension.

The strengths of the present study were the number of patients, the presence of two matched control groups with different degrees of hyperparathyroidism and the analysis of multiple nutritional parameters associated with PEW and malnutrition. Heavy smoking and alcohol drinking could impact nutritional status, but these data were either missing or incomplete in the medical records in most patients. The missing nPCR data prior to 2016 also prevented an accurate assessment of dietary protein intake especially among patients in cohort 2.

In conclusion, maintenance HD patients with severe hyperparathyroidism showed nutritional impairment which improved considerably after parathyroidectomy. These findings should emphasize the importance of early and proper management of hyperparathyroidism in patients with ESKD.

## Data availability statement

The original contributions presented in the study are included in the article/Supplementary material, further inquiries can be directed to the corresponding author.

## Ethics statement

The studies involving human participants were reviewed and approved by Faculty of Medicine, Ramathibodi Hospital, Mahidol University. Written informed consent for participation was not required for this study in accordance with the national legislation and the institutional requirements.

## Author contributions

SD conceived and designed the study. SD, SS, SK, and RS acquired the data. SD analyzed and interpreted the data. SD, RS, YW, and JT wrote the manuscript. All authors contributed to the article and approved the submitted version.

## Conflict of interest

The authors declare that the research was conducted in the absence of any commercial or financial relationships that could be construed as a potential conflict of interest.

## Publisher’s note

All claims expressed in this article are solely those of the authors and do not necessarily represent those of their affiliated organizations, or those of the publisher, the editors and the reviewers. Any product that may be evaluated in this article, or claim that may be made by its manufacturer, is not guaranteed or endorsed by the publisher.
