# Supplementary material for: Improvement of nutritional status after parathyroidectomy in patients receiving maintenance hemodialysis
Source: Front Med (Lausanne). 2023 Jul 6;10:1132566. doi: 10.3389/fmed.2023.1132566 (PMC10359897; doi:10.3389/fmed.2023.1132566)
Supplement: Supplementary file 1 [file Table_1.pdf]

Supplementary Table 1 Univariate linear regression of baseline factors on the change in body mass index (cohort 1)

Variables	≥ 10% increase in body mass index	
	Beta (95% Confidence Interval)	P-value
Age	-0.01 (-0.05, 0.03)	0.62
Sex (Male vs. Female)	0.36 (-0.66, 1.39)	0.48
Diabetes (Yes vs. No)	0.38 (-1.18, 1.94)	0.63
Baseline body mass index	-0.005 (-0.12, 0.11)	0.93
Dialysis vintage	0.001 (-0.01, 0.01)	0.92
Baseline laboratory data		
Hemoglobin	-0.14 (-0.41,0.13)	0.32
Neutrophil-to-lymphocyte ratio	0.003 (-0.3,0.31)	0.98
Albumin	-0.17 (-0.29, -0.04)	0.009
Creatinine/Body Surface Area	-0.005 (-0.009, -0.001)	0.02
Log parathyroid hormone	3.35 (1.33, 5.36)	0.001
Parathyroidectomy (Yes vs. No)	2.86 (0.81, 4.9)	0.006
