# Supplementary material for: Improvement of nutritional status after parathyroidectomy in patients receiving maintenance hemodialysis
Source: Front Med (Lausanne). 2023 Jul 6;10:1132566. doi: 10.3389/fmed.2023.1132566 (PMC10359897; doi:10.3389/fmed.2023.1132566)
Supplement: Supplementary file 2 [file Image_1.pdf]

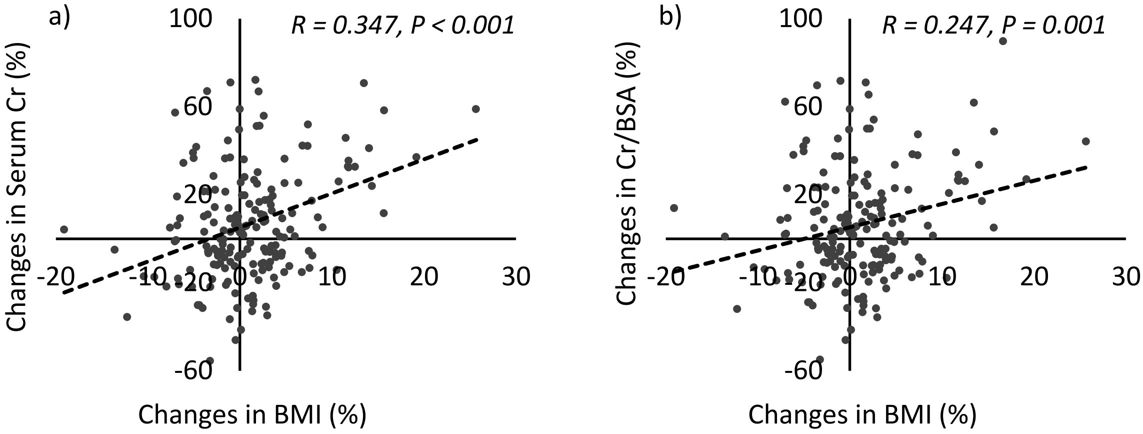


**Supplementary Figure 1** Pearson's correlation of the changes in body mass index from baseline (cohort 1) with
a) Changes in serum creatinine b) Changes in serum creatinine/body surface area
